# Functional input and membership characteristics in the accuracy of machine learning approach for estimation of multiphase flow

**DOI:** 10.1038/s41598-020-74858-4

**Published:** 2020-10-20

**Authors:** Meisam Babanezhad, Ali Taghvaie Nakhjiri, Mashallah Rezakazemi, Azam Marjani, Saeed Shirazian

**Affiliations:** 1 Institute of Research and Development, Duy Tan University, Da Nang, 550000 Vietnam; 2 Faculty of Electrical – Electronic Engineering, Duy Tan University, Da Nang, 550000 Vietnam; 3 Department of Petroleum and Chemical Engineering, Science and Research Branch, Islamic Azad University, Tehran, Iran; 4 Faculty of Chemical and Materials Engineering, Shahrood University of Technology, Shahrood, Iran; 5 Department for Management of Science and Technology Development, Ton Duc Thang University, Ho Chi Minh City, Vietnam; 6 Faculty of Applied Sciences, Ton Duc Thang University, Ho Chi Minh City, Vietnam; 7 Department of Chemical Sciences, Bernal Institute, University of Limerick, Limerick, Ireland; 8 Laboratory of Computational Modeling of Drugs, South Ural State University, 76 Lenin Prospekt, Chelyabinsk, Russia 454080

**Keywords:** Energy science and technology, Mathematics and computing

## Abstract

In the current study, Artificial Intelligence (AI) approach was used for the learning of a physical system. We applied four inputs and one output in the learning process of AI. In the learning process, the inputs are space locations of a BCR (bubble column reactor), which are *x*, *y*, and *z* coordinate as well as the amount of gas fraction in BCR. The liquid velocity is also considered as output. A variety of functions were used in learning, such as *gbellmf* and *gaussmf* functions, to examine which functions can give the best learning. At the end of the study, all of the results were compared to CFD (computational fluid dynamics). A three-dimensional (3D) BCR was used in this research, and we studied simulation by CFD as well as AI. The data from CFD in a 3D BCR was studied in the AI domain. In AI, we tuned for various parameters to achieve the best intelligence in the system. For instance, different inputs, different membership functions, different numbers of membership functions were used in the learning process. Moreover, the meshless prediction was used, meaning that some data in the BCR have not participated in the learning, and they were predicted in the prediction process, which gives us a special capability to compare the results with the CFD outcomes. The findings showed us that AI can predict the CFD results, and a great agreement was achieved between CFD computing nodes and AI elements. This novel methodology can suggest a meshless and multifunctional AI model to simulate the turbulence flow in the BCR. For further evaluation, the ANFIS method is compared with ACOFIS and PSOFIS methods with regards to model’s accuracy. The results show that ANFIS method contains higher accuracy and prediction capability compared with ACOFIS and PSOFIS methods.

## Introduction

Gas–liquid–solid and gas–liquid reactors as bubble columns are utilized in numerous pharmaceutical, biotechnological, and chemical industries. Moreover, other multiphase procedures are used as a result of their operative mixing and heat and mass transfer features between various phases using comparable energy related to vessels with stirred tanks^1,2^. Tiny bubbles are created through dispersing the gas phase within bubble column reactors in the liquid phase, continuously utilizing a tool for distributing the gas. Numerous operating circumstances affect the flow pattern, including the geometry, the gas flow rate governing the flow trend, and the gas distributors controlling the gas's spatial distribution and determining the primary bubble size distribution. Moreover, the bubble–bubble interactions and existence of turbulence and the complex interaction between operating circumstances result in complex flow structures and various flow trends dominating the flow’s hydrodynamic features. Hence, it is essential to comprehend the instantaneous flow systems completely.

Computational Fluid Dynamics (CFD) simulation was extensively developed over decades as a powerful computational method for predicting the instantaneous flow structures and mean flow profiles^3,4^. Though, it is still difficult to simulate the dynamics of mesoscale coherent flow structures in large-scale BCRs via traditional CFD simulations since fully resolving the dynamic behavior requires to compute the longer physical time (more than 1000 s) and finer grid resolution (almost 6.5 mm in radial, and 30 mm in the axial directions)^5^. Mentioning the simulation of longer physical time with finer grids requires time. However, a transient simulation of about several 100 s of physical time on relatively coarser grids is completed within several weeks or months of computational time^6,7^. This lower computational efficiency is caused by the restricted performance of traditional computer hardware, indeed, Central Processing Units (CPU) as well as the computational algorithm, including complex iterations in mathematical models and fluid solvers, and the incompatibility between computational algorithm and computer hardware in particular^8^. Therefore, coordination of the above issues is raised to effectively enhance computation speed and meet the process industry and chemical's practical need.

The phase-volume-averaged models, such as mixture or two-fluid models, are more feasible, although Direct Numerical Simulation (DNS)^9,10^ or Eulerian–Lagrangian model^11,12^ are more accurate and unaffordable. However, the mixture model is much simpler owing to the relatively weak coupling within phase equations and gives reasonable predictions for BCRs^13^. This simplicity in the model structure becomes more appropriate for computation acceleration with Graphics Processing Units (GPU). Hence, it is possible to obtain reasonable simulation accuracy and make the compatible model structure with GPU-accelerated parallel computation^14,15^. Nevertheless, numerical issues and convergence problems exist in all computing techniques that are sometimes very time-consuming.

Some examples of soft computing approaches in the literature include evolutionary algorithms, adaptive neuro-fuzzy inference system (ANFIS), neural networks support vector machines, and simulated annealing to simulate the physics and predict the flow of a bubble column reactor. ANFIS is more interesting in this regard by its capability for training complex relationships^16–18^. Many powerful and practical techniques have been produced by artificial intelligence to overcome the difficulties in different fields and solve complicated problems in the real world. Numerous researchers have employed artificial intelligence (AI) technology as a result of high-speed operation, its easy use, and acceptable accurateness not requiring to comprehend the physical issues. Azwadi et al.^19^ utilized the ANFIS technique for predicting the flow and temperature in a cavity. They performed the simulation of heat transfer behavior in a two-dimensional system considering different Reynolds numbers. They found that the flow fields and temperature are trained accurately predicted by the ANFIS algorithm in significantly less time. Machine learning methods are recently widely used to link to computational fluid dynamics to better predict the turbulent kinetic spectrums, gas hold-up, the liquid flow pattern, and other flow features in the BCR^20^. By this combination, a great framework is created to map the results or provide multiple input/output parameters^21,22^.

Researchers have conducted successful research about the prediction of different physical processes, such as multiphase flow, bubbly flow, and thermal analysis^23^. They have also tried to unlock the complicated processes, such as multiphase flow, the interaction between gas and liquid, and the interphase characteristics of the dispersed and continuous phases. They have mainly concentrated on the prediction of processes, based on experimental or numerical results^24,25^. They have also proposed different ways to train, test, validate, and then predict the processes and datasets. In addition to this analysis, they have illustrated the full intelligence flow pattern against the numerical flow pattern. However, machine learning methods have an excellent ability to understand the significant parameters and connectivity between input and output parameters. The level of complexity for each input parameter has not been thoroughly investigated. Alternatively, output parameters, such as flow characteristics (i.e., gas fraction, fluid velocity, and thermal distributions) or turbulence properties, solved by numerical methods, have not thoroughly been used in the input dataset. This study will address some of these research gaps. It will try to have more input parameters in the training process, a combination of geometry input parameters (location of computing points/nodes), and flow properties solved by CFD. Additionally, the complexity and connectivity between datasets are investigated to understand more about the process's input and output parameters.

In particular, in the current research paper, a 3D BCR is modeled via CFD, and the data from CFD is used in the training stage of the AI, and also used in the prediction stage via fuzzy logic. This BCR is 3D, and the data from *x*, *y*, and *z*-direction are trained, and data from solving the Navier–Stokes equation, which is air volume fraction, is also utilized in the training process of our data. Water liquid velocity is predicted as an output, and all of the models are studied with different functions. The effect of each input is tested in the accuracy of the model.

## Computational methodology

### Geometrical structure

A bubble column reactor (BCR) was studied at ambient temperature (23 °C) and atmospheric pressure in the current research. The arrangement of ring sparger orifices was orderly at fixed gaps in the termination of the multiphase domain. The bubble is detached based on the separation of the spherical bubbles that have clear shape distinction. Besides, it is further assumed that the bubbles possess minimum strike, with occurring the least breakup as well. Altogether, the above openings create a homogeneous flow regime with typical uniform bubble sizes in the multiphase flow domain.

### CFD simulations

Eulerian CFD technique is applied to model the gas bubble and liquid interaction in the multiphase framework, representing the approximation of gas and liquid volume fraction and not the interphase between two phases. The Eulerian model formulation, designated in this CFD research, is based on momentum transport equations and ensemble-averaged mass for the gas and liquid, respectively. As indicated below, the momentum transfer equations and continuity are denoted for the Euler–Euler multiphase framework, respectively (solved for the bubbles and liquid phases separately)^26^:

1 $$\frac{\partial }{{\partial {\text{t}}}}\left( {\rho_{{\text{k}}} \epsilon_{{\text{k}}} } \right) + \nabla \left( {\rho_{{\text{k}}} \epsilon_{{\text{k}}} {\text{u}}_{{\text{k}}} } \right) = { }0$$

where *ϵ*_*k*_ and *u*_*k*_ show the volume fraction of phase *k* and average velocity, respectively. To discretize the conservation equations, the control volume approach is employed in this numerical study. To solve the fluid flow issues in the CFD, some existing solution approaches include finite difference^27^, Lattice Boltzmann, and finite volume^26,28^. The finite volume discretization method is the most powerful procedure in which CFX is based. The momentum formulation for liquid phases and gas phase can be defined as^26^:

2 $$\frac{\partial }{{\partial {\text{t}}}}\left( {\rho_{{\text{k}}} \epsilon_{{\text{k}}} {\text{u}}_{{\text{k}}} } \right) + \nabla \left( {\rho_{{\text{k}}} \epsilon_{{\text{k}}} {\text{u}}_{{\text{k}}} {\text{u}}_{{\text{k}}} } \right) = - \nabla \left( {\epsilon_{{\text{k}}} \tau_{{\text{k}}} } \right) - \epsilon_{{\text{k}}} \nabla {\text{p}} + \epsilon_{{\text{k}}} \rho_{{\text{k}}} {\text{g}} + {\text{M}}_{{{\text{I}},{\text{k}}}}$$

The stress term of phase *k* in the above equation is represented as below^26^:

3 $$\tau_{{\text{k}}} = - \mu_{{{\text{eff}},{\text{k}}}} \left( {\nabla {\text{u}}_{{\text{k}}} + \left( {\nabla {\text{u}}_{{\text{k}}} } \right)^{{\text{T}}} - \frac{2}{3}{\text{I}}\left( {\nabla {\text{u}}_{{\text{k}}} } \right)} \right)$$

In Eq. 3, *μ* denotes effective liquid viscosity, consisting of 3 terms: turbulence viscosity, molecular viscosity, and viscosity based on bubble stimulated turbulence^26^:

4 $$\mu_{{{\text{eff}},{\text{L}}}} = \mu_{{\text{L}}} + \mu_{{{\text{T}},{\text{L}}}} + \mu_{{{\text{BI}},{\text{L}}}}$$

The operative viscosity of gas is written as:

5 $$\mu_{{{\text{eff}},{\text{G}}}} = \frac{{\rho_{{\text{G}}} }}{{\rho_{{\text{L}}} }}\mu_{{{\text{eff}},{\text{L}}}}$$

Sato and Sekoguchi^29^ suggested that two terms influence the turbulent shear stress in bubble flow. First, the inherent liquid turbulence does not depend on bubbles' relative motion in the liquid phase. Second, the extra liquid turbulence term created with bubble stirring^26^:

6 $$\mu_{BI,L} = \rho_{L} C_{\mu ,BI} \epsilon_{G} d_{B} \left| {u_{G} - u_{L} } \right|$$

The total interfacial force is the final term in the momentum transfer equation as defined below^26^:

7 $${\text{M}}_{{{\text{I}},{\text{L}}}} = - {\text{M}}_{{{\text{I}},{\text{G}}}} = {\text{M}}_{{{\text{D}},{\text{L}}}} + {\text{M}}_{{{\text{TD}},{\text{L}}}}$$

The above-mentioned total interfacial forces represent the turbulent distribution and drag force with neglecting the virtual mass and lift. The interphase momentum transfer between liquid phase and gas bubble because of drag force is presented below^26^:

8 $${\text{M}}_{{{\text{D}},{\text{L}}}} = - \frac{3}{4} \in_{{\text{G}}} \uprho_{{\text{L}}} \frac{{{\text{C}}_{{\text{D}}} }}{{{\text{d}}_{{\text{B}}} }}\left| {{\text{u}}_{{\text{G}}} - {\text{u}}_{{\text{L}}} } \right|\left( {{\text{u}}_{{\text{G}}} - {\text{u}}_{{\text{L}}} } \right)$$

In Eq. (8), the parameters *C*_*D*_ and *d*_*B*_ refer to the drag coefficient and bubble diameter, respectively. Diameters of 0.44 and 4 mm are chosen for the bubble diameter and drag coefficient, respectively, as recommended previously^5^ by experimental outcomes and numerical conditions of Plfeger and Becke^5^. According to earlier researches^30,31^, the turbulent dispersion force model is applied for the present CFD examinations to increase the flow field prevision against the walls. The above model, developed by Lopez de Bertodano^32^, is based on interplay and molecular movement analogy. That estimates a turbulent diffusion of the bubbles by the liquid eddies and is expressible as^26^:

9 $${\text{M}}_{{{\text{TD}},{\text{L}}}} = - {\text{M}}_{{{\text{TD}},{\text{G}}}} = - {\text{C}}_{{{\text{TD}}}} \rho_{{\text{L}}} {\text{k}}\nabla \in_{{\text{L}}}$$

In Eq. (9), *C*_*TD*_ and *k* represent turbulent dispersion coefficient and liquid Turbulent Kinetic Energy (TKE), respectively. A variety of values for the turbulent dispersion coefficient have been proposed in the literature^26,30^.

Besides interfacial forces, it is essential to select an appropriate turbulence model to properly predict bubble column hydrodynamics^31,33^. *k*–*ε* turbulence model is used to approximate velocity distribution and eddy structure due to gas and liquid interaction. This model is very popular in industries and academia due to fast calculation compared to direct numerical simulation or large-eddy simulations^26,33^. The turbulent eddy viscosity for multiphase flow (the interaction between gas bubbles and liquid) is expressed as:

10 $$\mu_{{{\text{T}},{\text{L}}}} = \rho_{{\text{L}}} {\text{C}}_{\mu } \frac{{{\text{k}}^{2} }}{\varepsilon }$$

The energy dissipation rate (*ε*) and its turbulent kinetic energy (*k*) are computed using the principal equations as follows^26^:

11 $$\frac{\partial }{{\partial {\text{t}}}}\left( {\uprho_{{\text{L}}} \in_{{\text{L}}} {\text{k}}} \right) + \nabla \left( {\uprho_{{\text{L}}} \in_{{\text{L}}} {\text{u}}_{{\text{L}}} {\text{k}}} \right) = - \nabla \left( { \in_{{\text{L}}} \frac{{\mu_{{{\text{eff}},{\text{L}}}} }}{{\sigma_{{\text{k}}} }}\nabla {\text{k}}} \right) + \in_{{\text{L}}} \left( {{\text{G}} - \uprho_{{\text{L}}} \varepsilon } \right){ }$$

12 $$\frac{\partial }{{\partial {\text{t}}}}\left( {\uprho_{{\text{L}}} \in_{{\text{L}}} \varepsilon } \right) + \nabla \left( {\uprho_{{\text{L}}} \in_{{\text{L}}} {\text{u}}_{{\text{L}}} \varepsilon } \right) = - \nabla \left( { \in_{{\text{L}}} \frac{{\mu_{{{\text{L}},{\text{eff}}}} }}{{\sigma_{\varepsilon } }}\nabla \varepsilon } \right) + \in_{{\text{L}}} \frac{\varepsilon }{{\text{k}}}\left( {{\text{C}}_{\varepsilon 1} {\text{G}} - {\text{C}}_{\varepsilon 2} \rho_{{\text{L}}} \varepsilon } \right){ }$$

where *k* and *ε* are determined using their conservation equations.

Parameter *G* specifies the production of turbulent kinetic energy^26^:

13 $$G = \uptau_{{\text{L}}} :\nabla {\text{u}}_{{\text{L}}}$$

#### Grid

For the generation of computing domain in CFD study, hexahedral grids are generated throughout the multiphase domain. The meshes' non-uniform structure is copied in different levels of the reactor from sparger to bubble column surface zones. As the flow regime in this work is homogeneous flow with typical uniform spherical bubbles, we validated the gas hold-up in the reactor and liquid flow pattern with the existing dataset in literature for homogeneous flow regime. In the big difference between CFD outputs and experimental observations, we need to check CFD boundary conditions and numerical implementation. However, sometimes, in experimental observations, we cannot fully observe the bubble column reactor's flow behaviour due to experimental noise and errors.

### Adaptive-network-based fuzzy inference system (ANFIS)

ANFIS is a fuzzy-based simulation system for the precise prevision of the action of complex physical/chemical systems . Three dissimilar kinds of fuzzy rationale exist for which Sugeno and Takagi recommended if–then approach to be employed in the structure of ANFIS^34^. The function of the ith rule for different input parameters are defined as^35^:

14 $$w_{i} = \mu_{Ai} \left( X \right) \mu_{Bi} \left( Y \right)\mu_{ci} \left( Z \right)\mu_{Di} \left( {{\text{AVF}}} \right)$$

where *w*_*i*_ is the outcoming signal of the 2nd layer's node, and *μ*_*Ai*_, *μ*_*Bi,*_* μ*_*Ci*_ and *μ*_*Di*_ are incoming signals from MFs applied on inputs, x coordination (X), y coordination (Y), z coordination (Z) and air volume fraction (AVF), to the 2nd layer's node^35^.

Weight function for different patterns of prediction are described as:

15 $$\overline{{w_{i} }} = \frac{{w_{i} }}{{\sum \left( {w_{i} } \right)}}$$

In Eq. (15), $\overline{{w_{i} }}$ is named normalized firing strengths. The 4th layer used the structure of a consequent if–then rule suggested by Sugeno and Takagi^34^. Therefore, the node function may be defined as:

16 $$\overline{{w_{i} }} f_{i} = \overline{{w_{i} }} \left( {p_{i} X + q_{i} Y + r_{i} Z + S_{i} AVF + t_{i} } \right)$$

where *p*_*i*_, *q*_*i*_, *r*_*i,*_, *s*_*i*_ and *t*_*i*_ are the if–then rules' parameters termed consequent parameters^35^.

## Results and discussion

**Figure 1:**
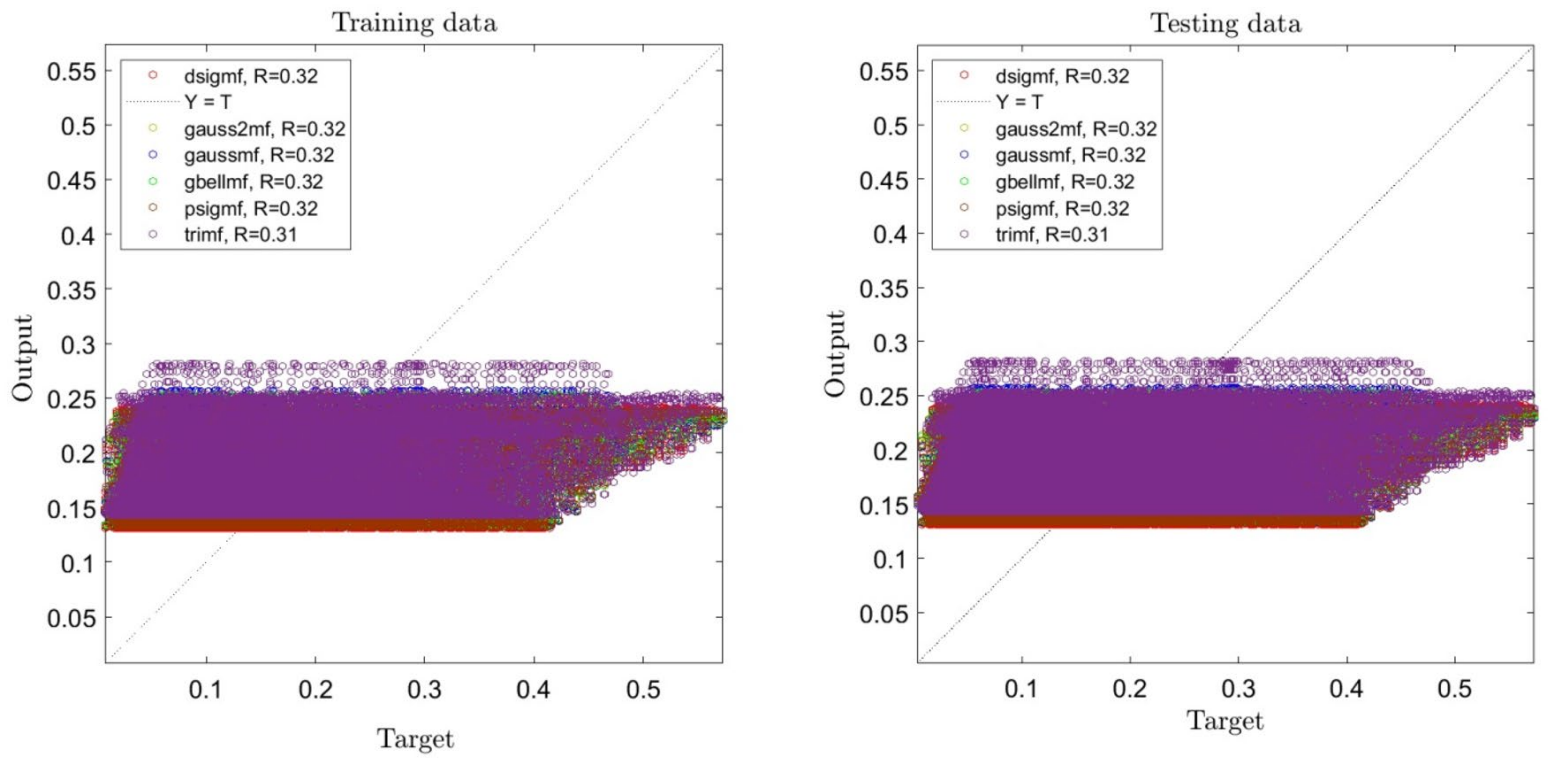
ANFIS training and testing processes using one input and different types of membership functions when number of membership functions is 2 for each input.

Sixty percent of data was applied for learning process in the current study, and the remaining data was studied in the testing process. As mentioned before, different functions with a variety in number were studied in learning, and we consider the best signaling for the intelligence of the system in our study, and for achieving the best intelligence in the system, we optimize the system. As shown in Fig. 1, one input was used for all the functions and the membership function in the system. Using one input leads to having very low intelligence in the system. When one input is used in training, generally, the data does not have the prediction capability.

**Figure 2:**
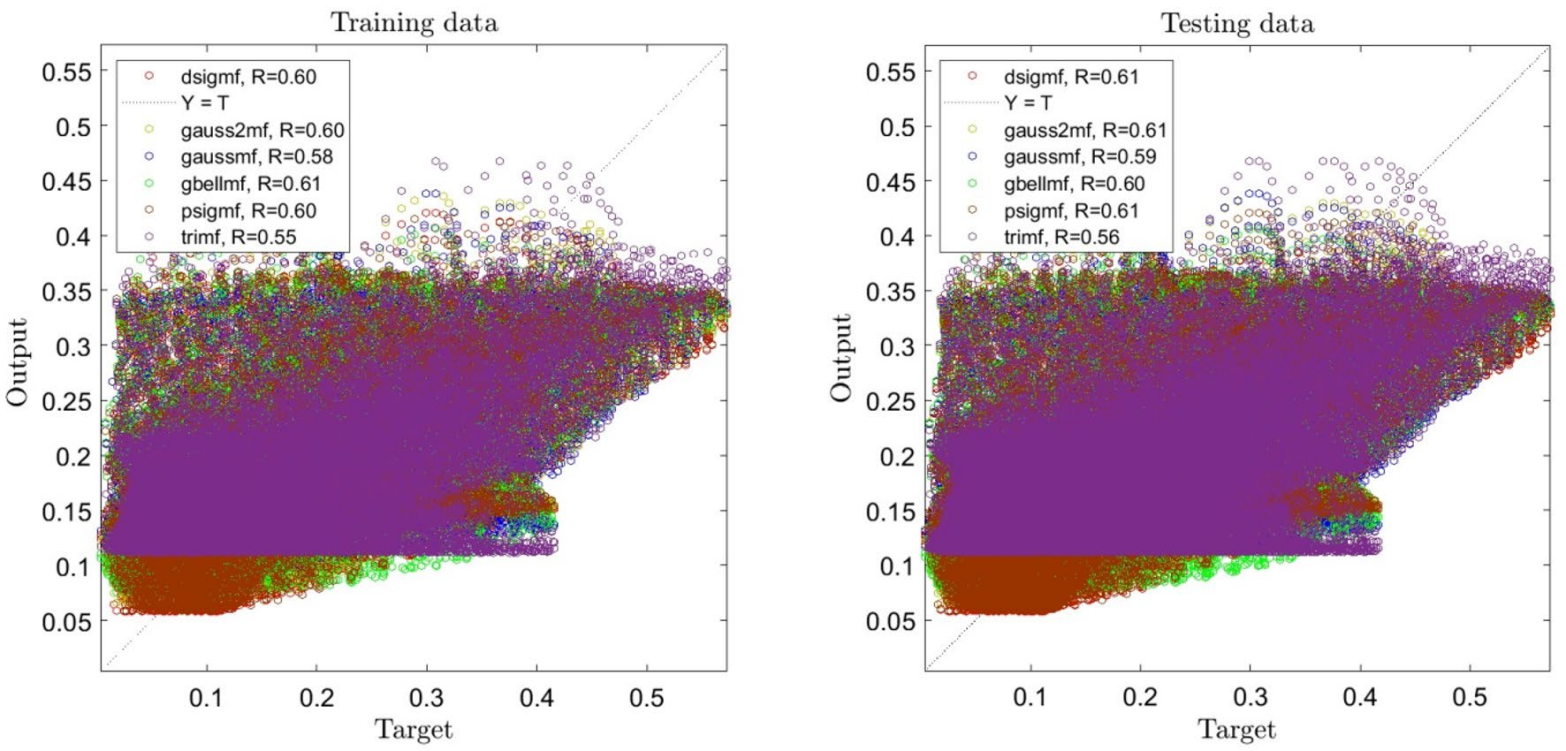
ANFIS training and testing processes using two inputs and different types of membership functions when number of membership functions is 2 for each input.

**Figure 3:**
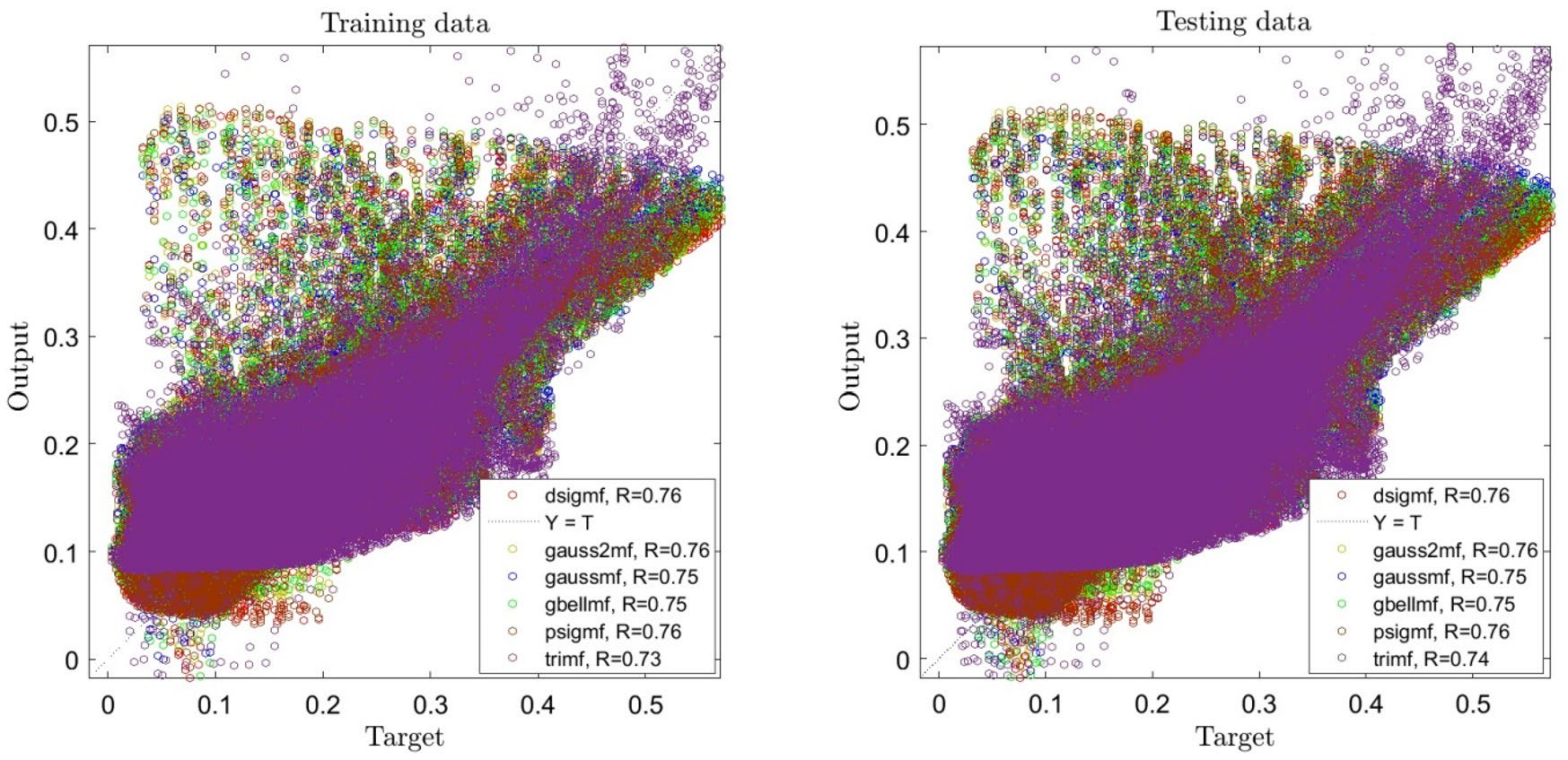
ANFIS training and testing processes using three inputs and different types of membership functions when number of membership functions is 2 for each input.

By enhancing the number of inputs, a significant change in the error domain is seen. As shown in Fig. 2, the system's accuracy increases compared to 1 input, and a slight difference can be seen when the functions in the system change, but the positive point is that by increasing the inputs in our system, the system reaches a good intelligence. On the other hand, Fig. 3 shows that when the number of inputs is increased to 3, the system's R reaches 0.76, and we can see an escalating trend in the system. Analyzing the data shows us that the accuracy of the system stays steady when the functions change.

**Figure 4:**
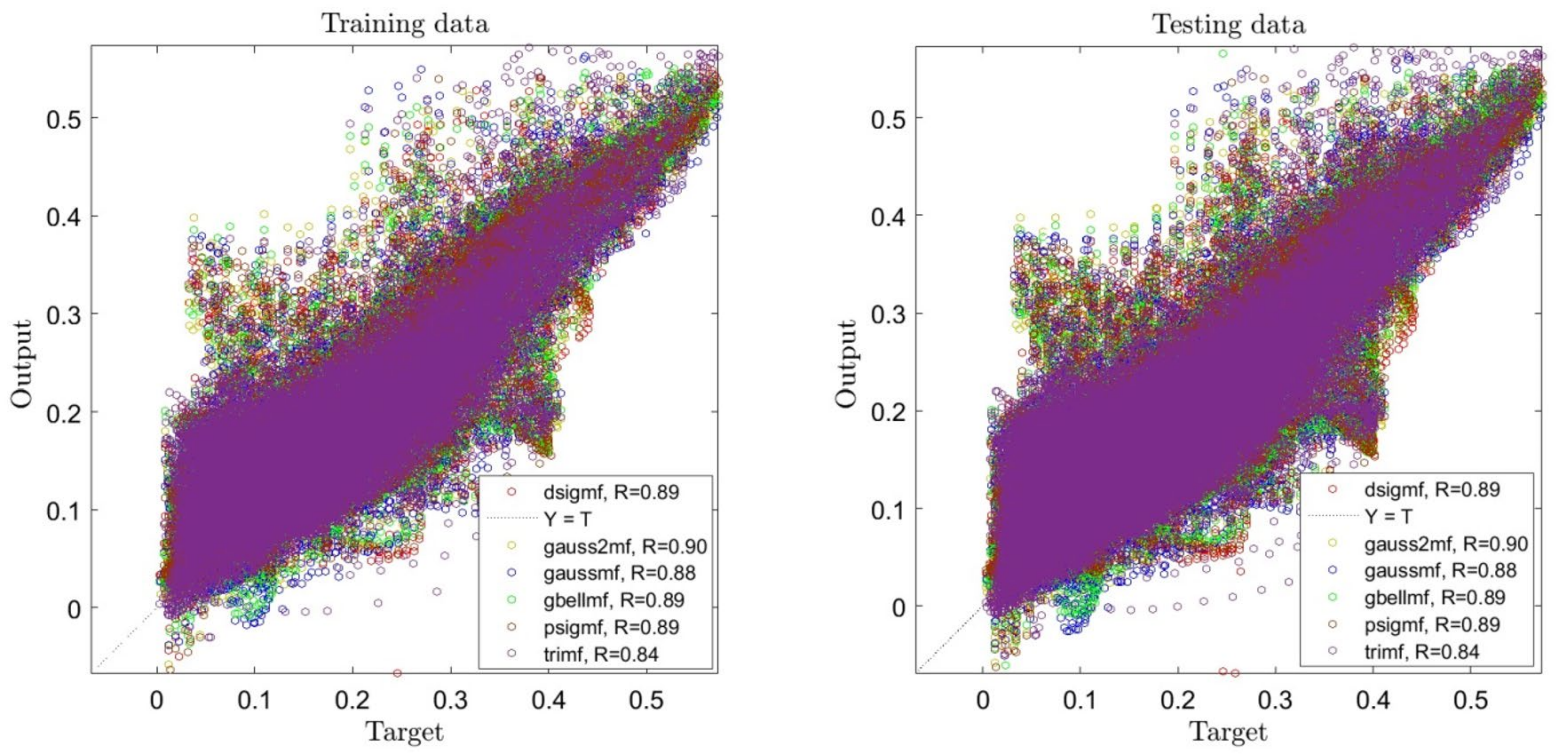
ANFIS training and testing processes using four inputs and different types of membership functions when number of membership functions is 2 for each input.

Figure 4 shows four inputs in learning, and a significant improvement is visible in the accuracy of the system, and R reaches 0.89, which is a suitable number, and even for some functions, R reaches 0.9.

**Figure 5:**
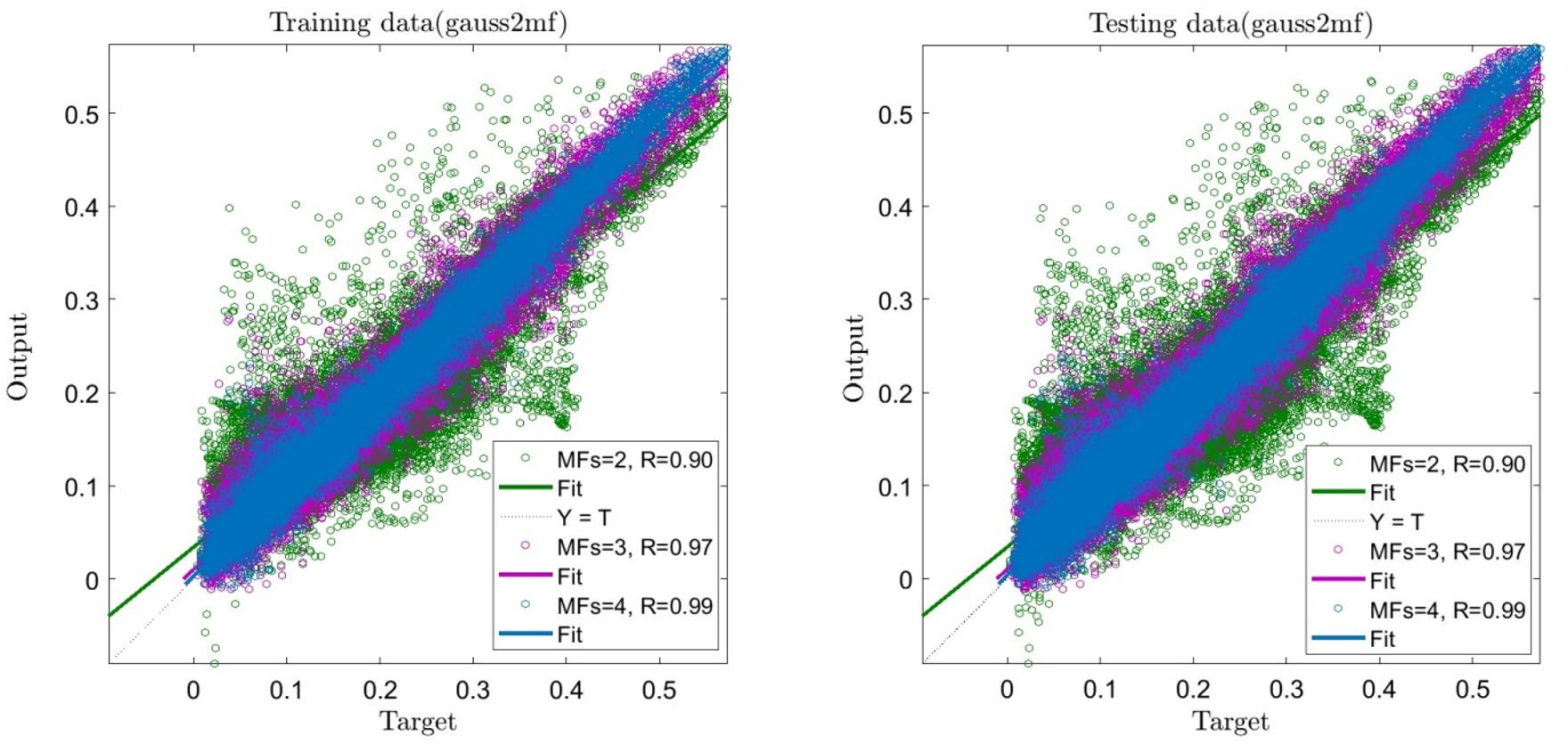
ANFIS training and testing processes using four inputs and *gaussmf* as the best type of membership function with changes in number of membership functions (2,3 and 4).

**Figure 6:**
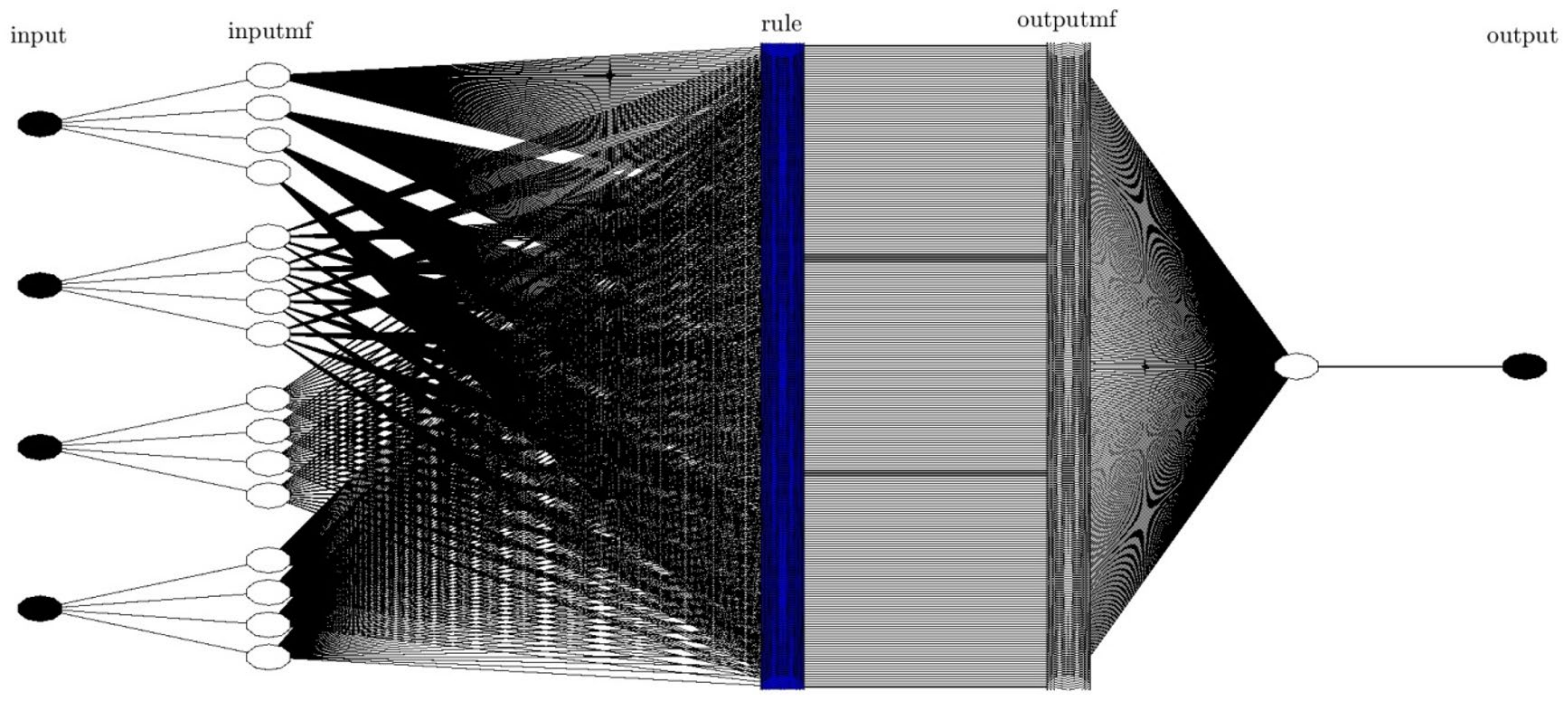
ANFIS structure in the best intelligence using four inputs and number of membership functions is 4 for each input with type of *gaussmf*.

A point worth mentioning about Fig. 5 is that the number of inputs is kept constant, and the membership functions changes. So, when the membership functions increase, the system sends a suitable intelligent signal; therefore, the system can be considered the absolute intelligence of the BCR. The same figure shows that membership functions 2, 3, and 4 were considered, and R for the mentioned membership functions are 0.9, 0.97, 0.99 successively. As is evident, the membership function for number 4 is a very high number. Figure 6 shows the neural networks for four inputs. As shown, a bulky neural network is created according to the high number of membership functions and the system's inputs.

**Figure 7:**
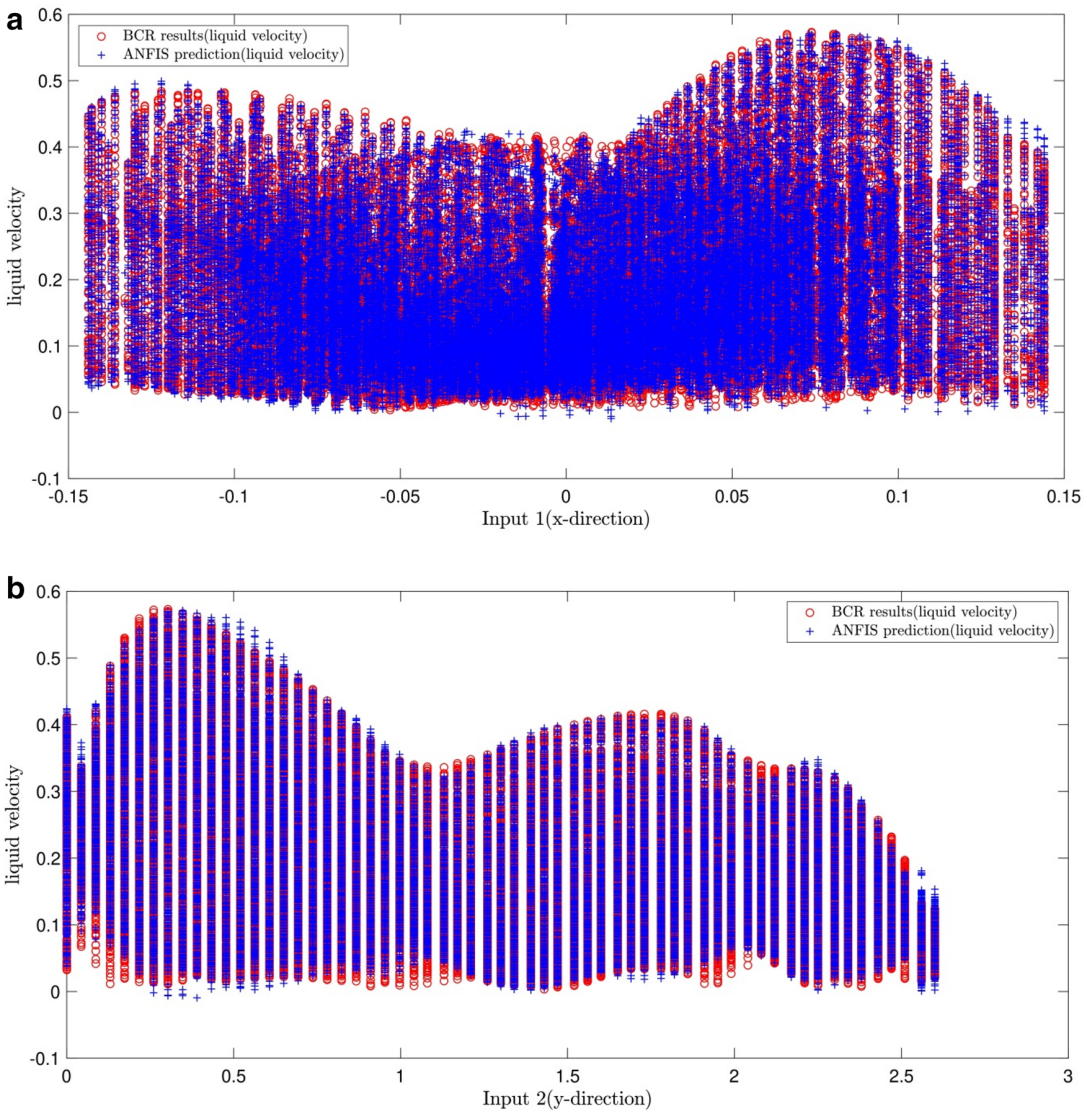

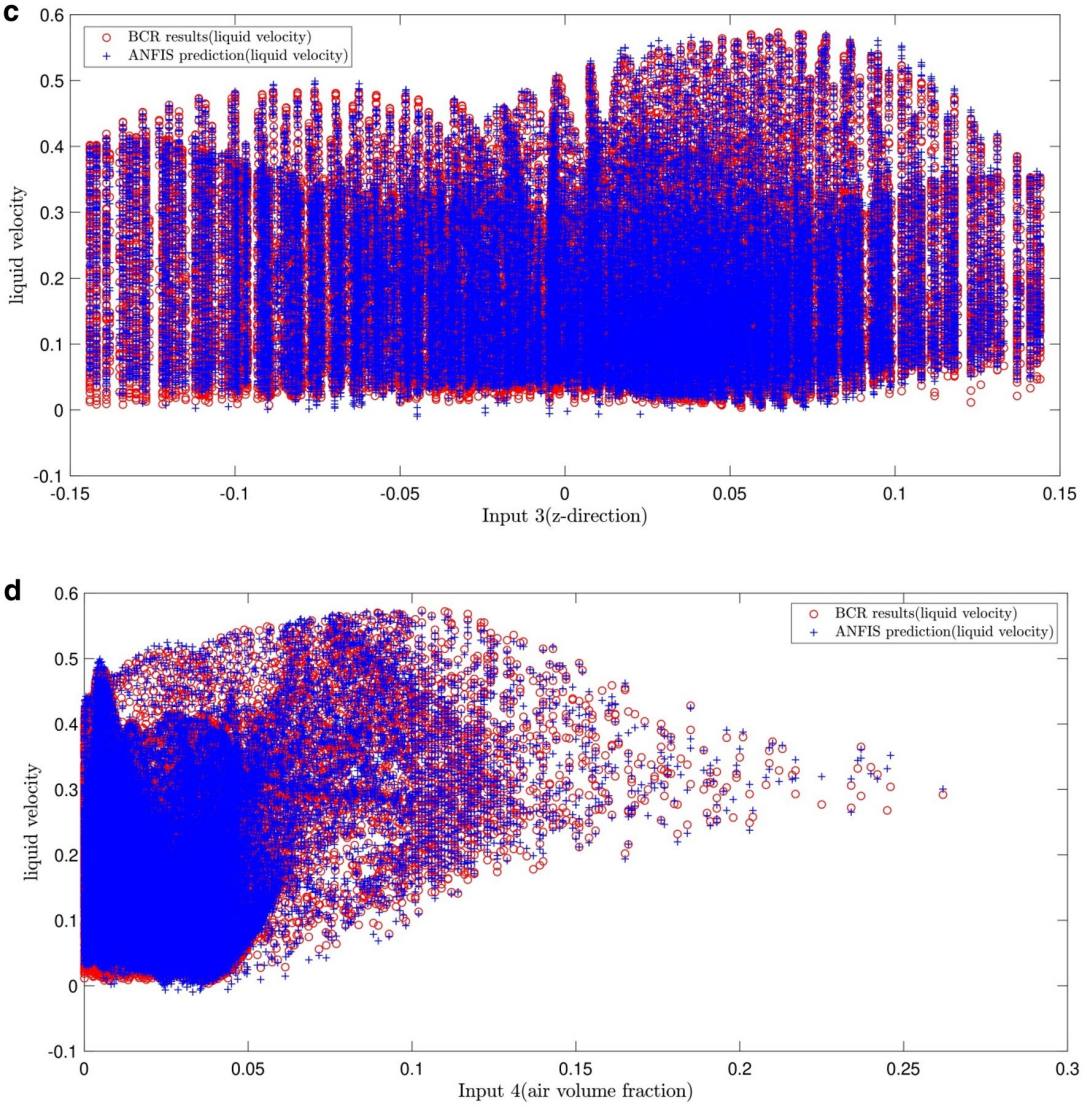
(**a**) Validation of ANFIS prediction (liquid velocity) and BCR simulated results (liquid velocity), considering first input (x-direction). (**b**) Validation of ANFIS prediction (liquid velocity) and BCR simulated results (liquid velocity), considering second input (y-direction). (**c**) Validation of ANFIS prediction (liquid velocity) and BCR simulated results (liquid velocity), considering third input (z-direction). (**d**) Validation of ANFIS prediction (liquid velocity) and BCR simulated results (liquid velocity), considering forth input (air volume fraction).

Figure 7a–d show the number of membership functions and inputs keeps in the best condition meaning that the former and the latter are kept 4. Also, the changes in the system show us their effects on the error by considering the inputs. As illustrated in Fig. 7a, the velocity of liquid results from the function of input 1, and the data from CFD and AI overlap each other.

**Figure 8:**
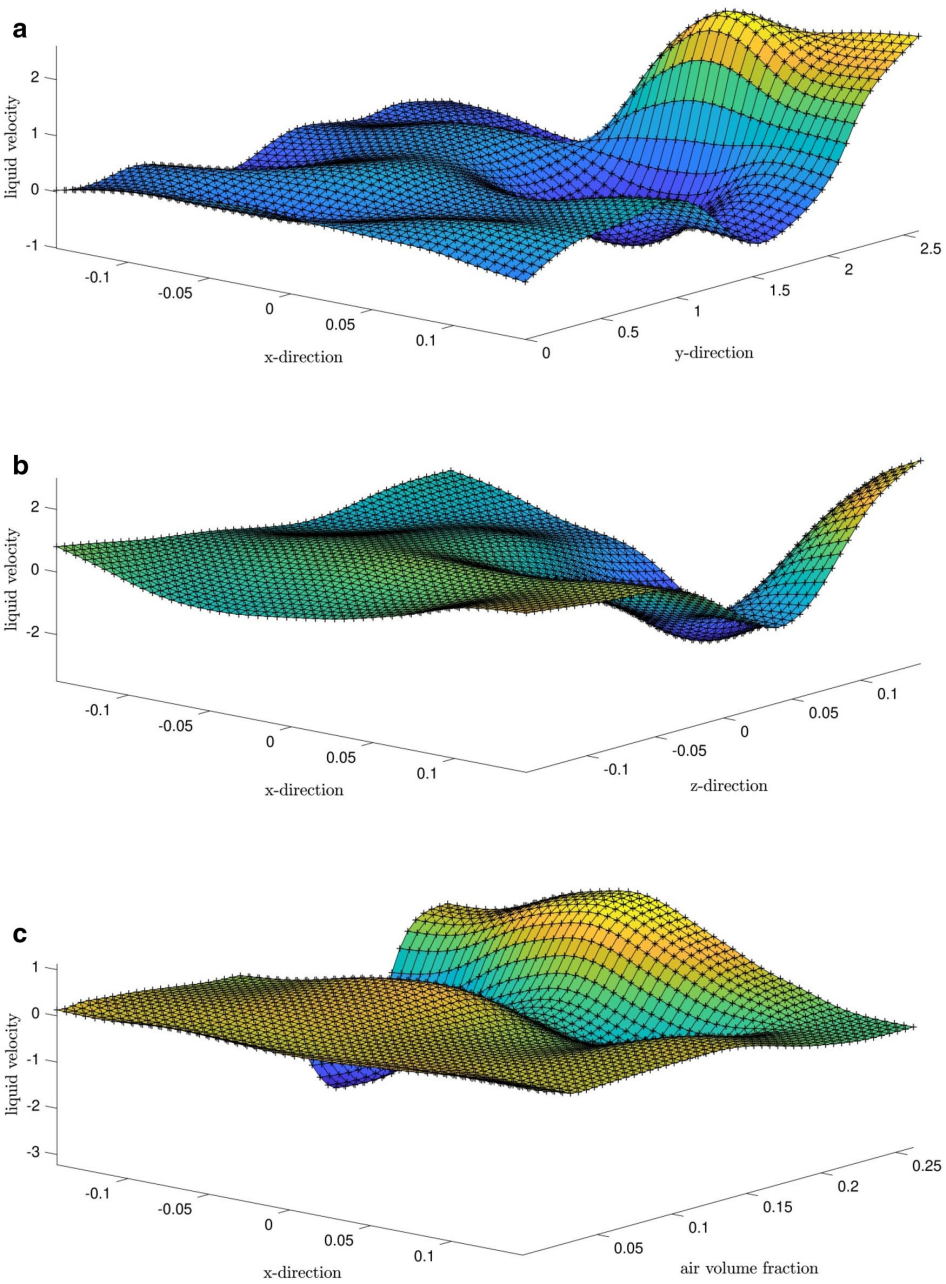

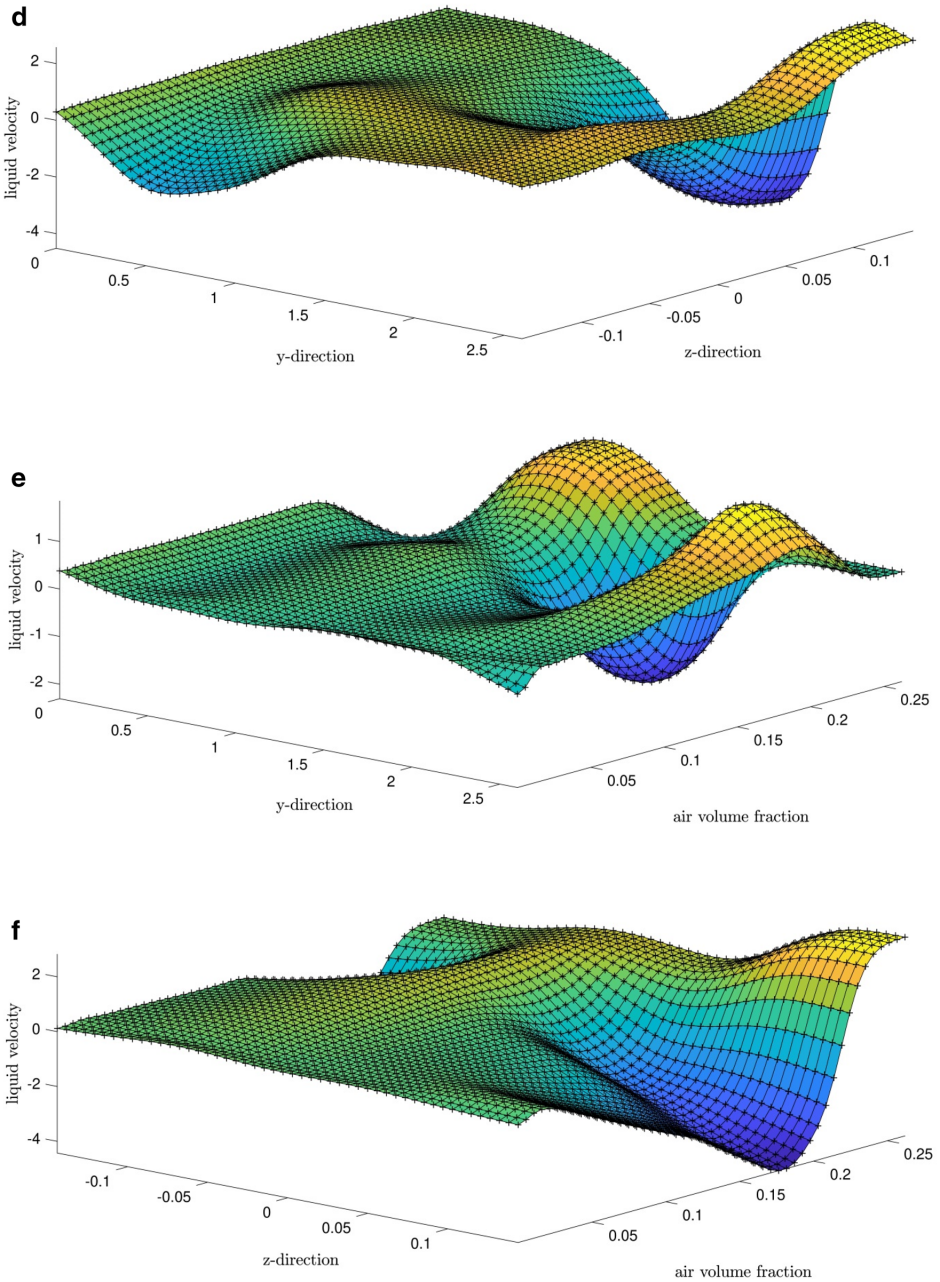
(**a**) Liquid velocity predicted surface with x-direction and y-direction as inputs. (**b**) liquid velocity predicted surface with x-direction and z-direction as inputs. (**c**) Liquid velocity predicted surface with x-direction and air volume fraction as inputs. (**d**) Liquid velocity predicted surface with y-direction and z-direction as inputs. (**e**) Liquid velocity predicted surface with y-direction and air volume fraction as inputs. (**f**) Liquid velocity predicted surface with z-direction and air volume fraction as inputs.

**Figure 9:**
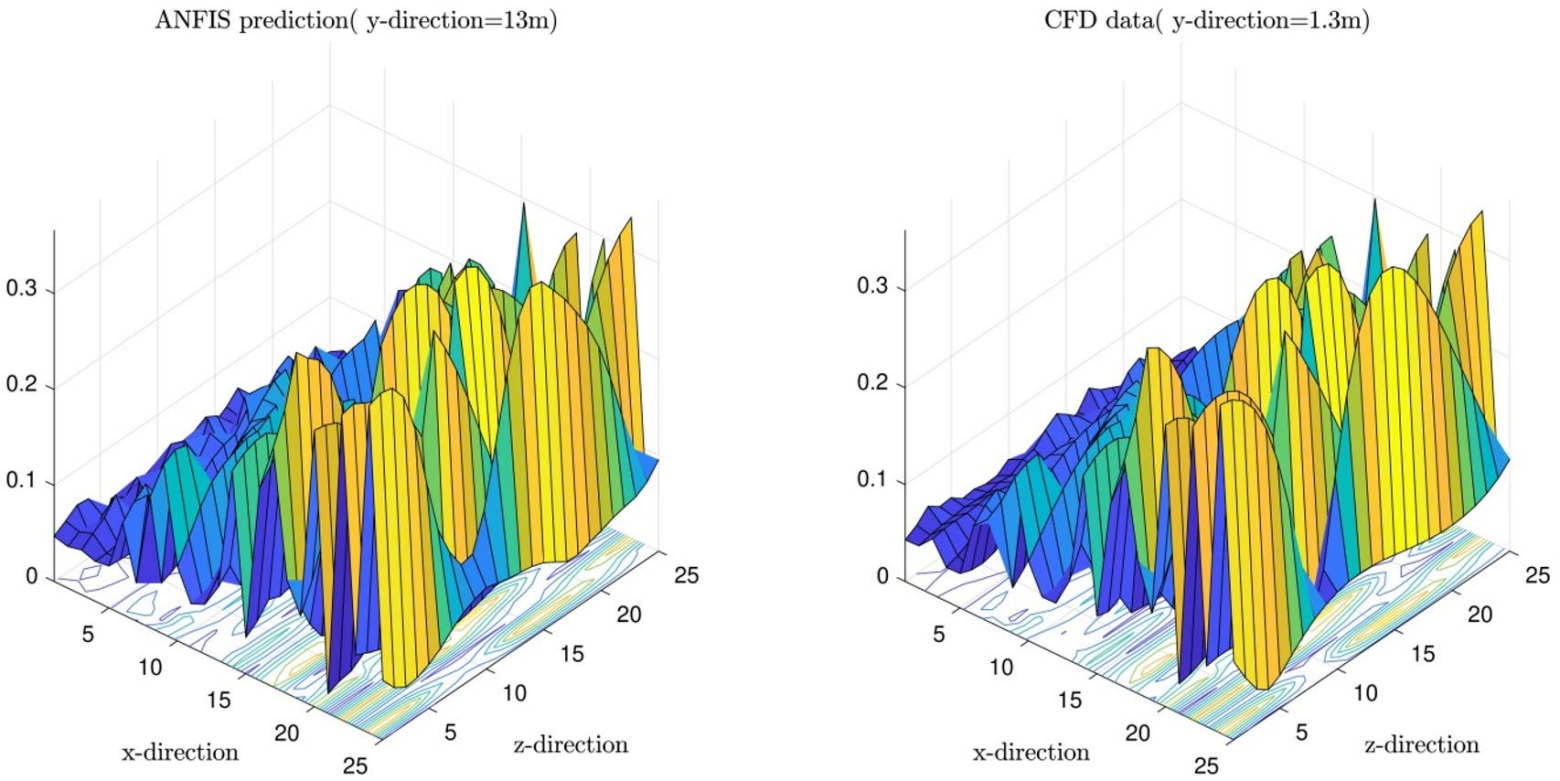
Comparison of CFD data and ANFIS prediction using data which was absent in learning processes (Y-direction = 1.3 m).

In Fig. 8a–f, we consider the 3D faces for the general prediction. Also, we consider inputs 1 and 2 with the output. As shown on top of the BCR, the highest amount of increase in the fluid velocity is seen. The fluid movement in the corner of the BCR is aggregated, which is shown in Fig. 8. Figure 9 compares the CFD and ANFIS data. As shown, AI and CFD patterns are generally similar. We used a full meshless method, and some data were not used in the AI, so AI could predict the data very intelligently, which overlaps with the CFD results.

**Figure 10:**
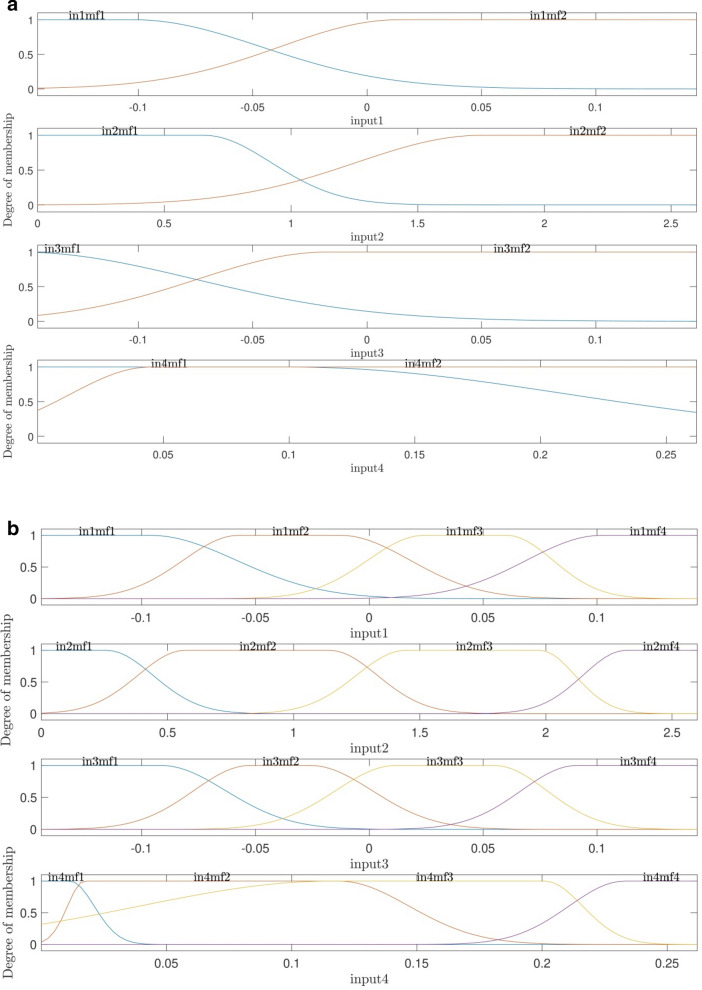
(**a**) Degree of membership using *guass2mf* as type of MFs when number of MFs for each input is 2. (**b**) Degree of membership using *guass2mf* as type of MFs, when number of MFs for each input is 4.

To understand more about the impact of the number of membership functions on the method's accuracy and prediction capability, the degree of membership functions were analyzed as a function of each input parameter. This analysis also showed the connectivity between inputs and outputs parameters and the level of input complexity. Figure 10a,b showed the degree of membership function as a function input parameters. First, two functions on each input were considered. The results showed that considering two membership functions in each input function could not cover the whole range of input parameters. In this regard, the number of membership functions increased in each input for Fig. 10b, the results illustrated that more functions could represent an input parameter in a better way.

**Figure 11:**
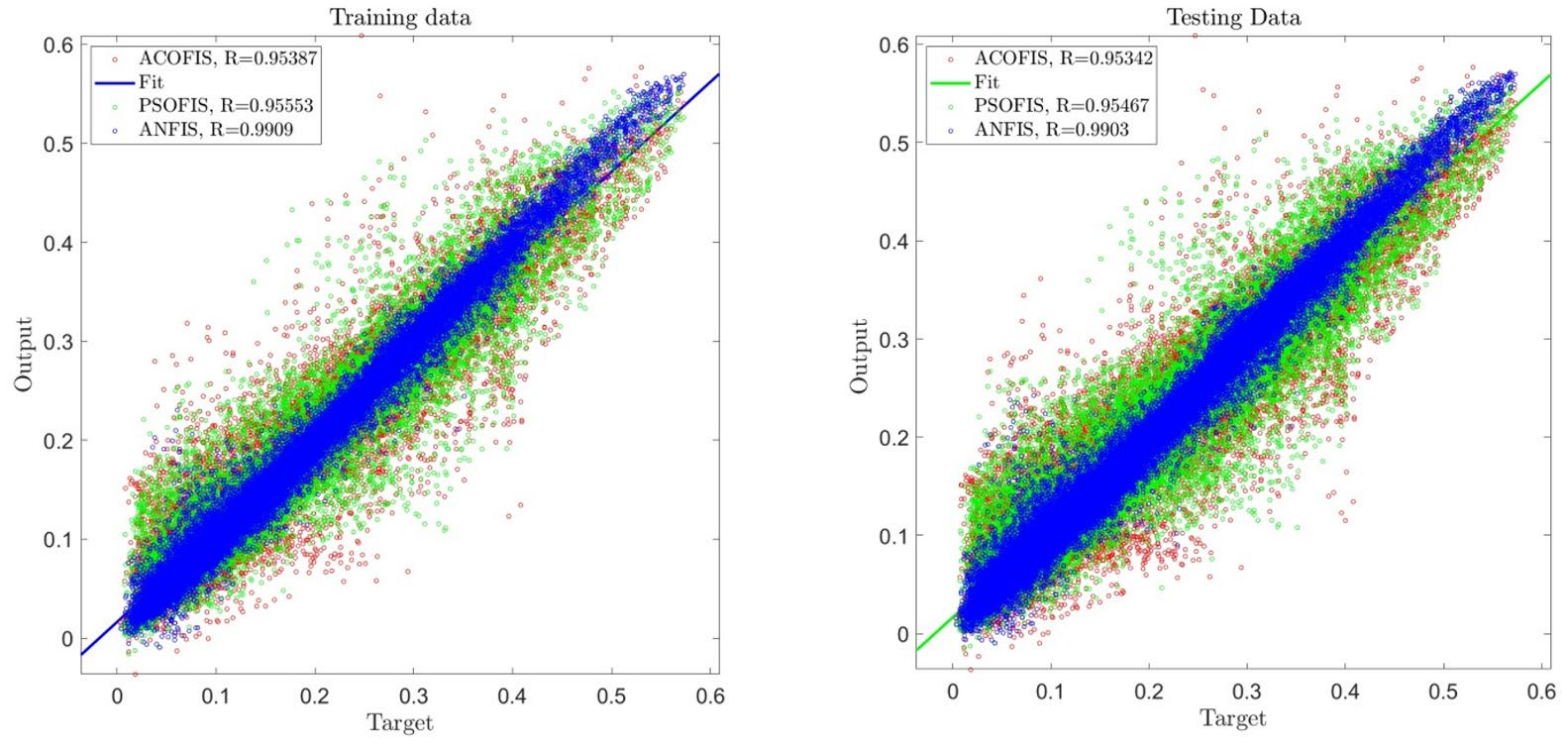
Regression plot of the best prediction using ANFIS, ACOFIS and PSOFIS methods.

**Figure 12:**
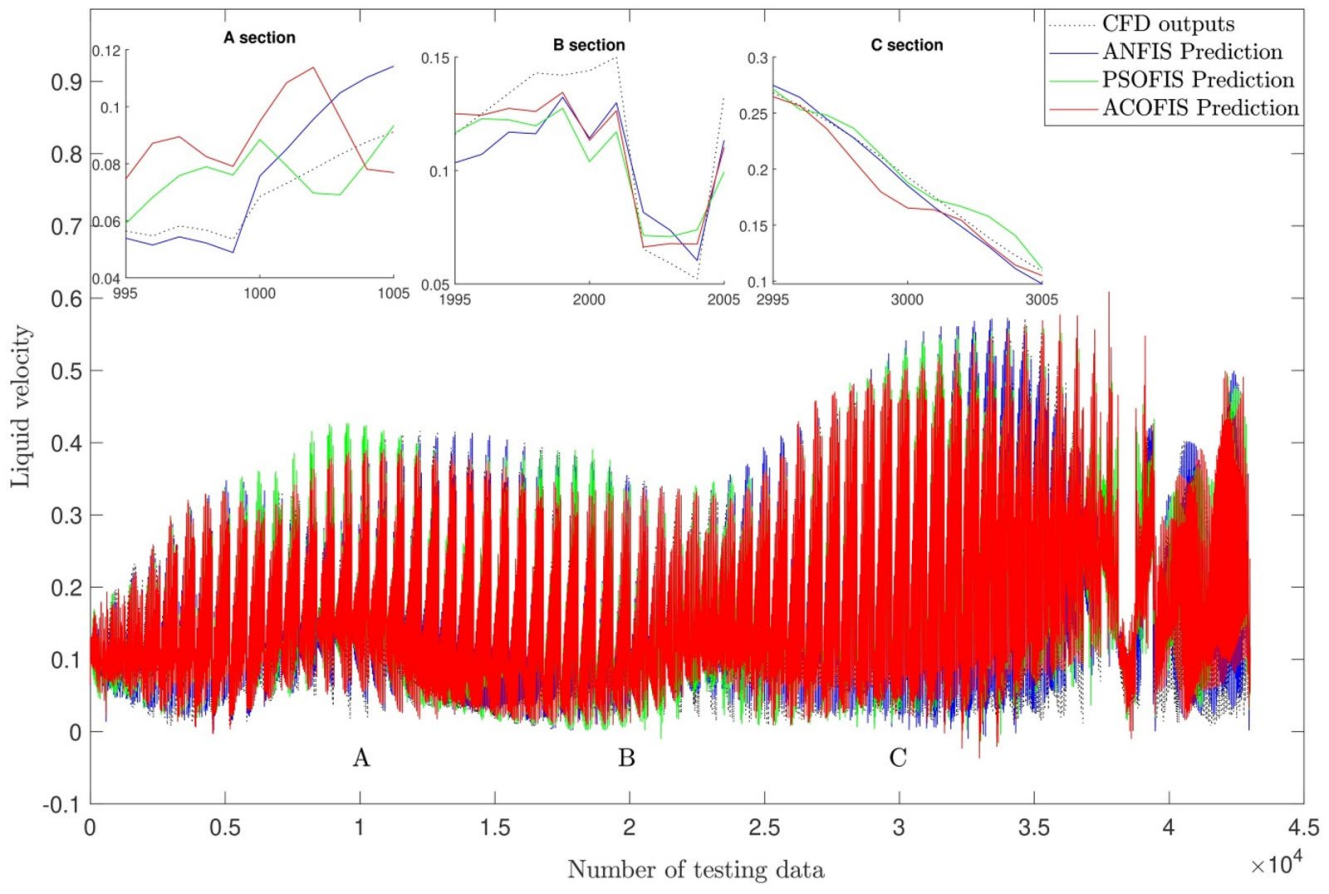
Comparison of predictions pattern by ANFIS, ACOFIS and PSOFIS methods.

Different learning algorithms, such as ACO and PSO methods, were considered in the training process for a better comparison between the current AI method with other traditional AI methods. All numerical conditions for machine learning methods are similar, such as the percentage of the training dataset, type of membership functions, and the number of numerical iterations (See Table 1). In this regard, the training accuracy level and the prediction capability were evaluated for the ANFIS method. Figure 11 showed that the ANFIS method's accuracy is higher than ACOFIS and PSOFIS methods with regards to the training and testing processes. For instance, the ANFIS method contained R > 0.99, while ACOFIS and PSOFIS methods were less than 0.96 for R criteria. Apart from the accuracy of this for the training and testing process, the behavior of this method in the prediction of the fluid flow pattern in the domain should be considered. Figure 12 compared the fluid flow pattern prediction for different machine learning and CFD methods. The results illustrated that there is a good agreement between the ANFIS method and CFD results, and this method of prediction can accurately estimate the flow pattern in the domain. For more analysis and error assessment, all training and testing process for the ANFIS, ACOFIS, and PSOFIS methods were compared together (see Table 2). The results show that the ANFIS method can accurately train datasets and then predict them. This method contained a minimal error with regards to $R, R^{2} , RSME$ and $STD$ evaluation analysis.

**Table 1:** Initial parameters of ANFIS, ACOFIS and PSOFIS methods.

Method	ANFIS	ACOFIS	PSOFIS
MaxIteration	70	70	70
Number of inputs	4	4	4
Number of P	60	60	60
Clustering type	Grid partition	Subtractive clustering	Subtractive clustering
Cluster influence range (CIR) as a parameter of subtractive clustering	–	0.15	0.15
FIS type	sugeno	sugeno	sugeno
Number of rules	256	122	125
Number of membership function for each input	4	122	125
Number of membership function for output	256	122	125
Type of membership function	*gauss2mf*	*gaussmf*	*gaussmf*

**Table 2:** Comparison of errors in different artificial intelligence algorithms.

Method	ANFIS	ACOFIS	PSOFIS
Train error MSE	0.000221471	0.001097562	0.001062102
Train error RMSE	0.014881887	0.033129476	0.032589912
Train error mean	4.50921E − 09	9.99085E − 18	− 0.000246356
Train error standard deviation (StD)	0.014882176	0.033130118	0.032589613
Train correlation coefficient (R)	0.9909	0.95387	0.95553
Test error MSE	0.000235801	0.001111123	0.00108232
Test error RMSE	0.015355813	0.033333514	0.032898639
Test error mean	− 3.06986E − 05	0.000190029	− 0.000200651
Test error standard deviation (StD)	0.015355961	0.03333336	0.03289841
Test correlation coefficient (R)	0.9903	0.95342	0.95467

## Conclusions

By analyzing the results, we understood that increasing the number of inputs, the system has an intelligent signal, and we can easily use AI in the three-dimensional BCR for the prediction of fluid flow and gas in the BCR. We modeled the three-dimensional BCR with CFD, and different hydrodynamic parameters in the BCR were simulated via the CFD method. Then they were inputted to the AI, and by changing the inputs in the AI system including changing in the number of membership function, the number of nodes, and different membership functions, we could reach an AI system which is fully intelligent. When the number of inputs increases to 4, the system shows an exemplary reaction, meaning that it reaches its best conditions. For evaluation of the current AI method, this method is compared with ACOFIS and PSOFIS methods regarding training accuracy, prediction capability, and pattern recognition. The method of the ANFIS showed a significant level of prediction compared with other AI methods, and it can accurately track the fluid flow pattern in the domain. The current study shows us that AI can be used in different flow regimes, and the relations among the flow regimes can be found with AI. But before finding the relations among the regimes, the system must reach its highest intelligence. The current prediction method can only distinguish between gas and liquid phases, which are based on the data-driven training. This method can only predict the process within the range of training. In this regard, this method cannot predict different flow regimes or phases outside the training datasets. The AI method can be an assistance tool besides numerical methods to understand the process better, find a significant parameter in the process, and optimize the process for many operating conditions.
